# Give me some sugar: a transporter responsible for sugar uptake from the rhizosphere identified in apple

**DOI:** 10.1093/plphys/kiad342

**Published:** 2023-06-14

**Authors:** Yana Kazachkova

**Affiliations:** Assistant Features Editor, Plant Physiology, American Society of Plant Biologists, Rockville, MD, USA; Department of Molecular Biology, Princeton University, Princeton, NJ 08817, USA

Apples (*Malus domestica* Borkh.) are one of the major fruit crops worldwide. Originally from central Asia east of the Caspian Sea, apples are now cultivated and consumed globally. Apple fruit quality is directly correlated with organic carbon content in the soil (Kai and Adhikari 2021). Below the ground, organic matter is decomposed into simple sugars that are reused by plants and soil microbes. However, it was unclear how plant roots can absorb sugar molecules from the rhizosphere and whether it is a passive diffusion or an active transporter-mediated process.

Evidence from Arabidopsis studies suggests that AtSTP1, a member of the HEXOSE TRANSPORTER (HT)/SUGAR TRANSPORT PROTEIN (STP) family, plays a leading role in hexose (namely glucose) transport in roots (Yamada et al. 2011). HT transporters are localized to the plasma membrane and predominantly transport hexoses against the concentration gradient (Sauer et al. 1990; Lee and Seo 2021). Although there is compelling data on the substrate specificity of HT transporters, the mechanisms of HT-mediated hexose transport and its effect on plant biomass and carbon allocation remains unknown.

In this issue of *Plant Physiology*, Xiaocheng Tian and colleagues identified an apple sugar transporter, MdHT1.2, which plays a role in glucose uptake from the rhizosphere by root epidermis cells (Tian 2023). The authors hypothesized that transporters similar to the AtSTP1 could participate in hexose uptake by apple roots. They analyzed gene expression profiles of all 29 putative *AtSTP1* homologs in the existing apple RNAseq datasets. *MdHT1.2* had the highest expression levels in root tissues and was upregulated by exogenous glucose treatment. Biochemical analysis and fluorescently tagged protein imaging showed that *MdHT1.2* was mainly expressed on the plasma membrane of the epidermis cells of fine roots. These data suggested that MdHT1.2 was a promising candidate protein that transports sugar molecules from the rhizosphere across the plasma membrane in the epidermis cells of apple roots.

To demonstrate that MdHT1.2 can transport sugars, the authors expressed it in HT-deficient mutant yeast strains that cannot use glucose or other hexoses as a carbon source. MdHT1.2 complemented the growth-deficient mutant phenotypes, indicating that MdHT1.2 could transport hexoses in yeast cells. To understand how hexose transport by MdHT1.2 would affect the carbon allocation on the whole plant level, the authors generated *MdHT1.2* overexpression and knock-down apple plant lines. Roots of overexpression plants had higher glucose levels, and their growth was promoted by exogenous glucose application. Overexpression plants also had a higher biomass compared with the wild-type and knock-down plants. Although the total biomass of overexpression plants was increased, the root-to-shoot ratio was lower, suggesting a possible change in photosynthetic carbon allocation from shoot to root compared with the wild type. To test this hypothesis, the authors performed ^13^C pulse labeling experiments. They fed the plants with ^13^CO_2_ and analyzed the ^13^C distribution pattern. ^13^C concentration in *MdHT1.2* overexpression apple roots was significantly lower compared with that in the wild type, while the enrichment of ^13^C in the aerial part of the plant was observed. The results suggest that higher glucose uptake by the roots caused more photosynthetic carbon retention in the aerial parts of the plants, leading to higher biomass accumulation and lower root-to-shoot ratios. Because the authors were using a constitutive promotor to drive the expression of *MdHT1.2*, it raises the question of whether heterologous expression of the transporter protein in green tissues could also partially influence the results of this experiment. It would be interesting to determine whether similar patterns of ^13^C distribution occur if the transporter is expressed at higher levels compared with the wild type but in a tissue-specific manner rather than in the whole plant.

Because apple trees have a long lifecycle, the authors used tomato plants to constitutively express *MdHT1.2* to study the role of MdHT1.2 in sugar accumulation in fruit. When grown in soil, supplemented with glucose, tomato fruits from the overexpression lines had significantly higher levels of glucose and other sugars, indicating that heterologous overexpression of *MdHT1.2* could positively regulate sugar accumulation in the fruit. To test whether the increase in sugar content could be explained by the amplified allocation of assimilated photosynthetic carbon to the fruit, the authors performed an elegant grafting experiment. Wild-type and *MdHT1.2* overexpressing lines were used as rootstocks and scions in all the possible rootstock/scion combinations. The resulting grafts were grown on medium with and without glucose supplementation. The presence of exogenous glucose in the media led to the increased fruit size, which was significantly more pronounced when the overexpression plant was used as a rootstock. However, the increase in fruit size was not accompanied by any changes in plant photosynthesis. To assess changes in photosynthetic carbon allocation, the authors performed ^13^C labeling experiments and discovered that no changes in carbon distribution occurred on the media without glucose. However, with glucose supplementation, when the overexpression plants were used as a rootstock, ^13^C partitioning to the fruit was increased. This indicates that transporter-mediated glucose uptake from the rhizosphere reduces the consumption of photosynthetic carbon by root tissues, leading to increased photosynthetic carbon retaining in aerial parts of the plant and therefore higher fruit sugar content (Fig.).

**Figure.**
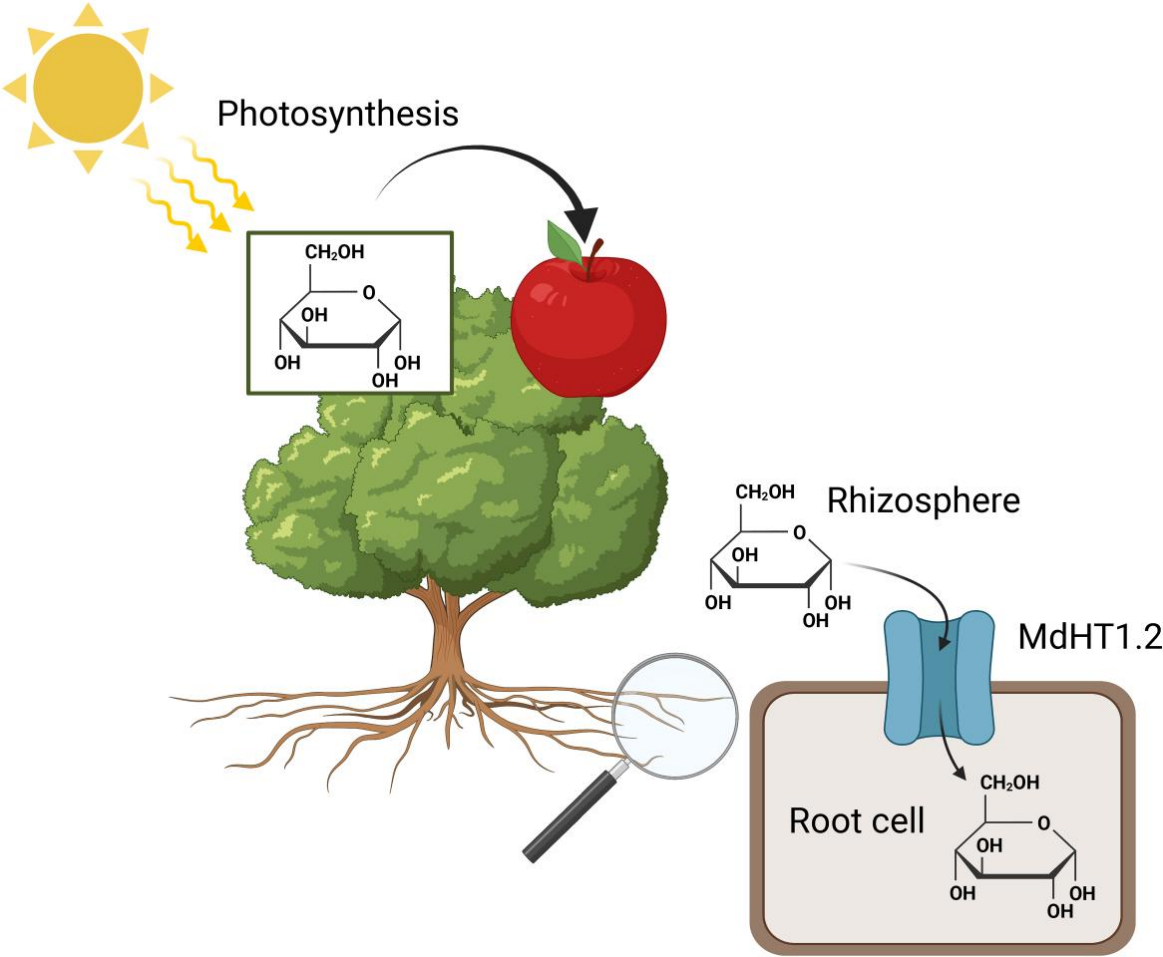
A model of MdHT1.2 function in the regulation of glucose allocation and fruit sugar accumulation. During photosynthesis, plants assimilate CO_2_ to produce sugars that are allocated to nonphotosynthetic organs, such as roots and fruit. When *MdHT1.2* is transporting sugar molecules (glucose as an example in the figure) from the rhizosphere into the root cells, less photosynthetic carbon is allocated for root growth and development. Hence there is an increase in assimilated sugar distribution to other reservoirs such as fruit. The figure was created in Biorender.

In the manuscript, the authors present a discovery of MdHT1.2, a root hexose transporter playing a role in glucose uptake from the rhizosphere. In transgenic apples and tomatoes, its overexpression changes the carbon allocation pattern between the roots and shoots of the plant, facilitating carbon partitioning to the fruit. It would be interesting to investigate the role of *MdHT1.2* homologs in other agriculturally important fruit-bearing crops and assess their potential as a breeding target for sweeter and larger fruit.
